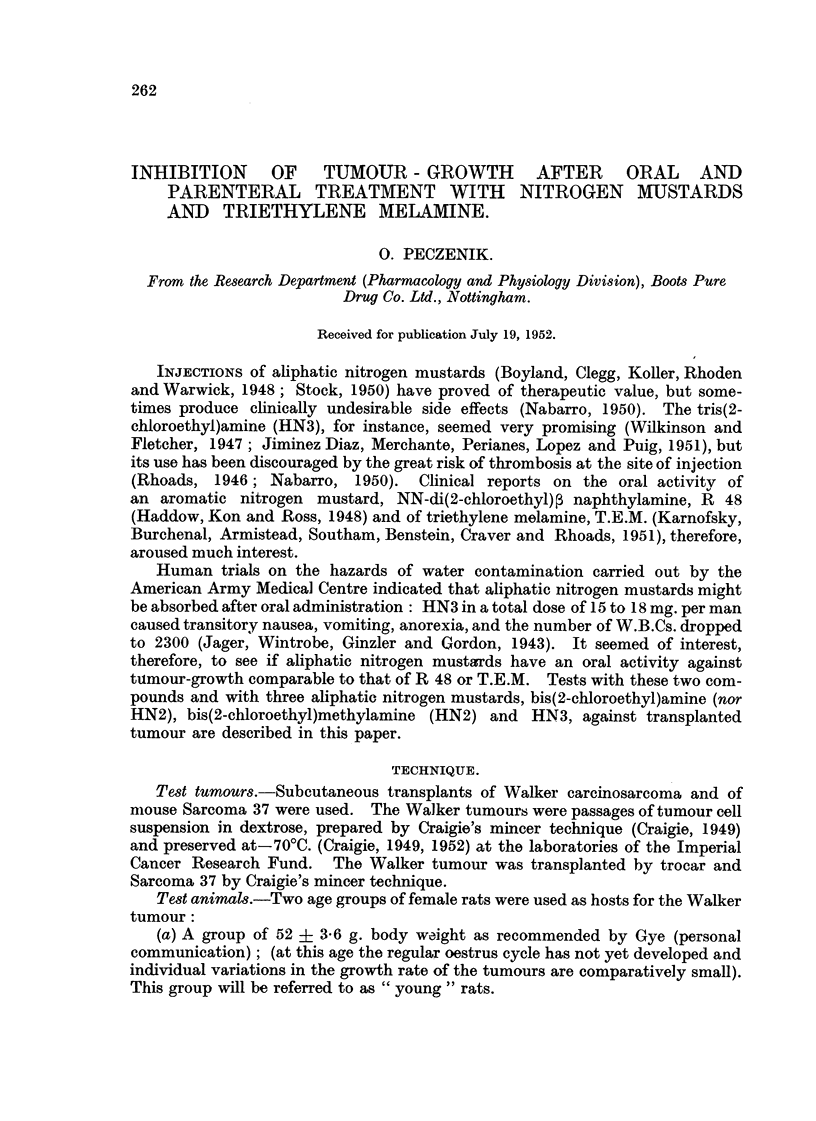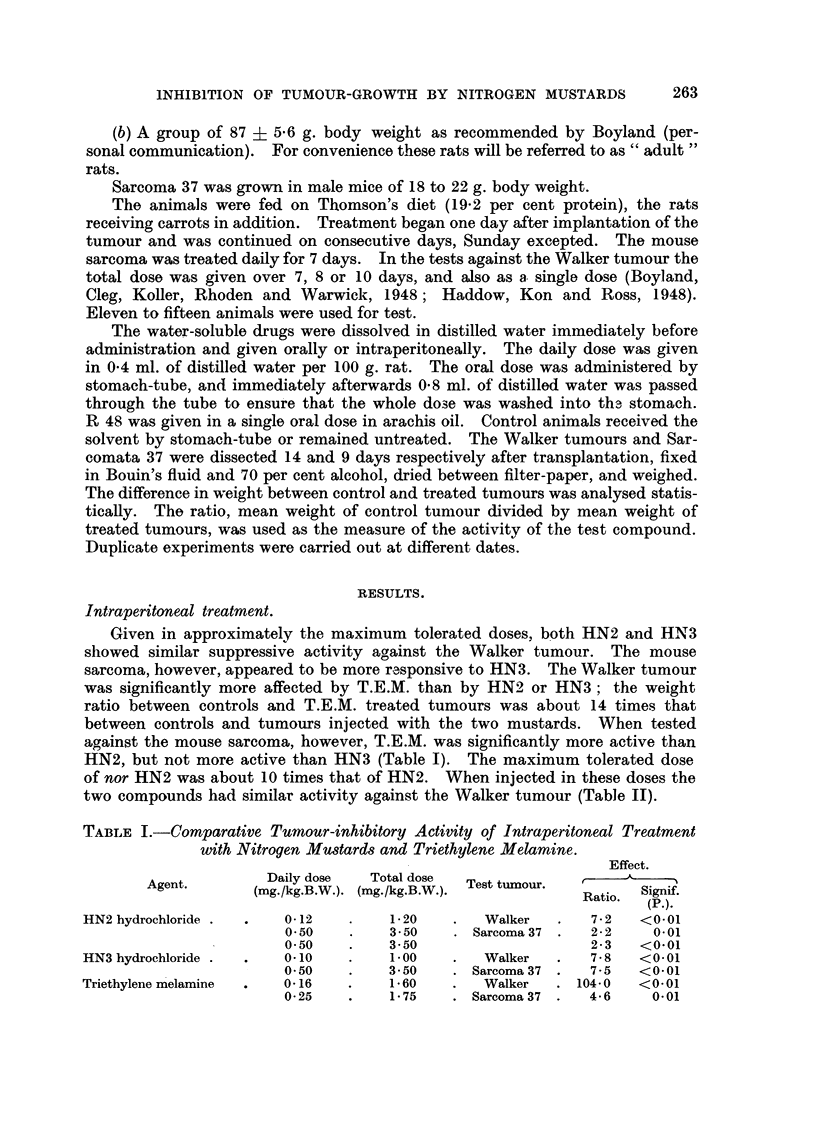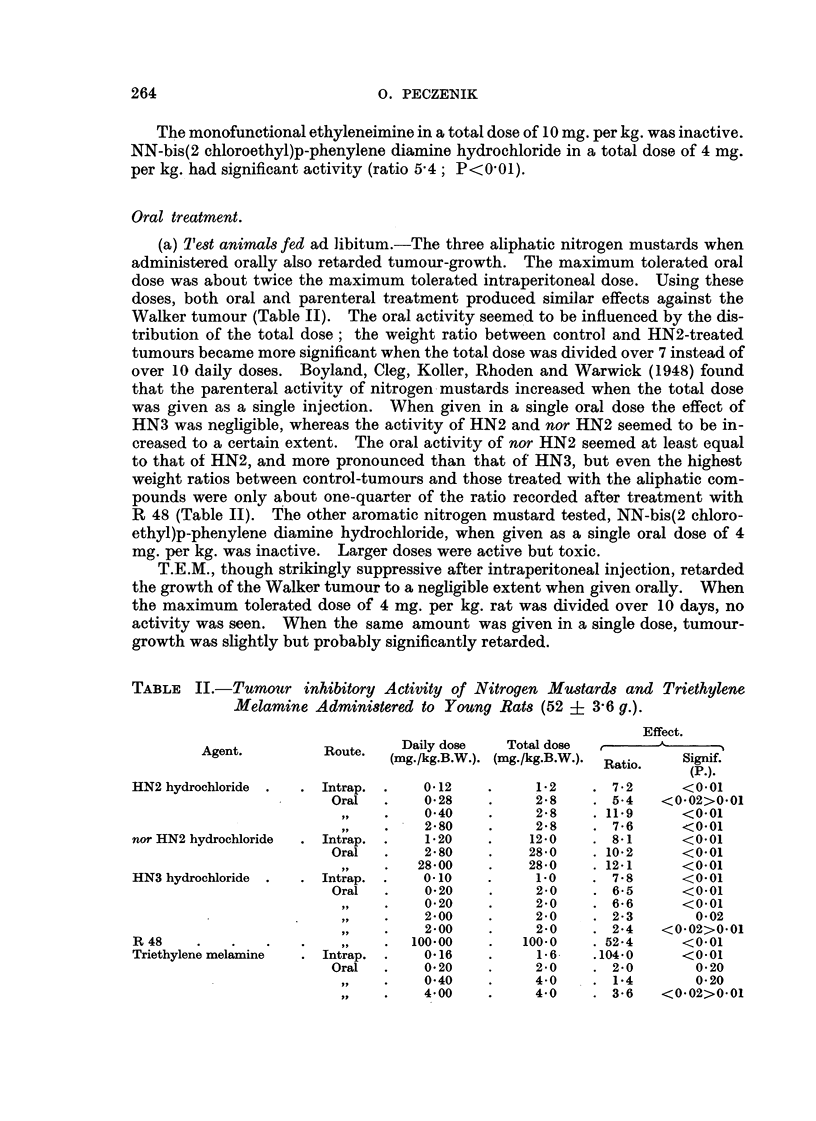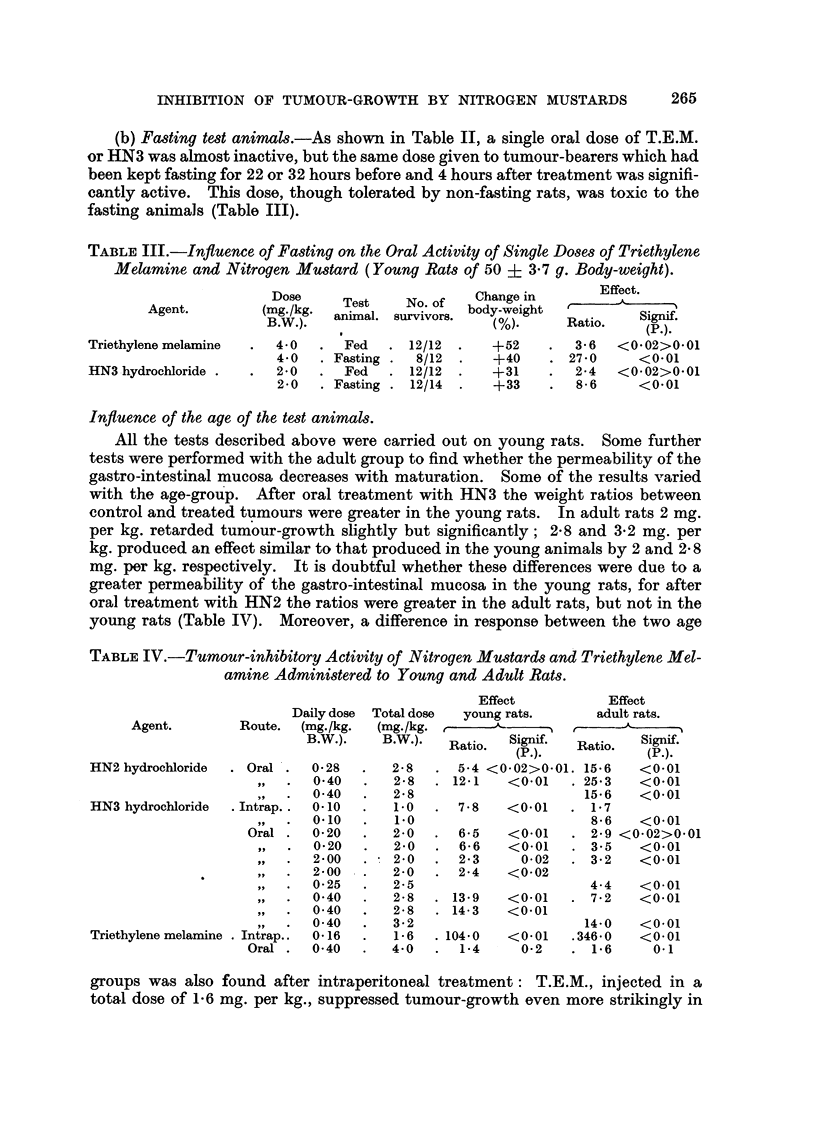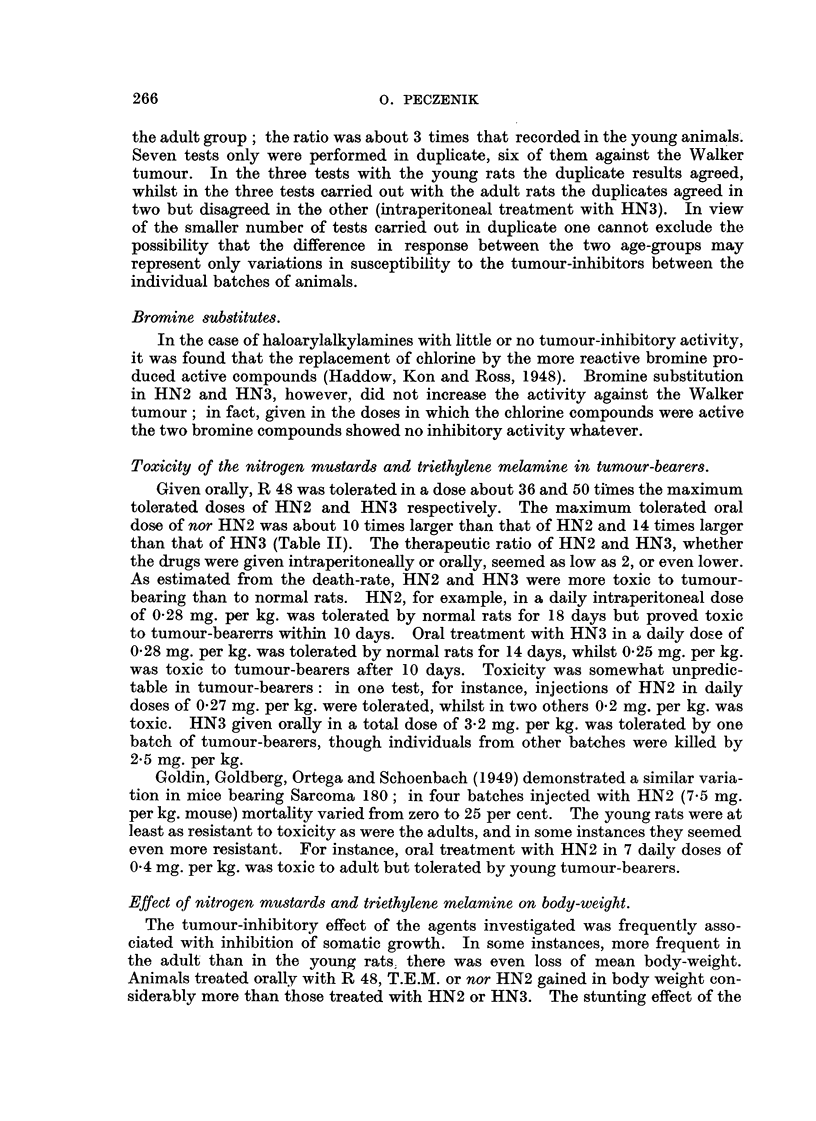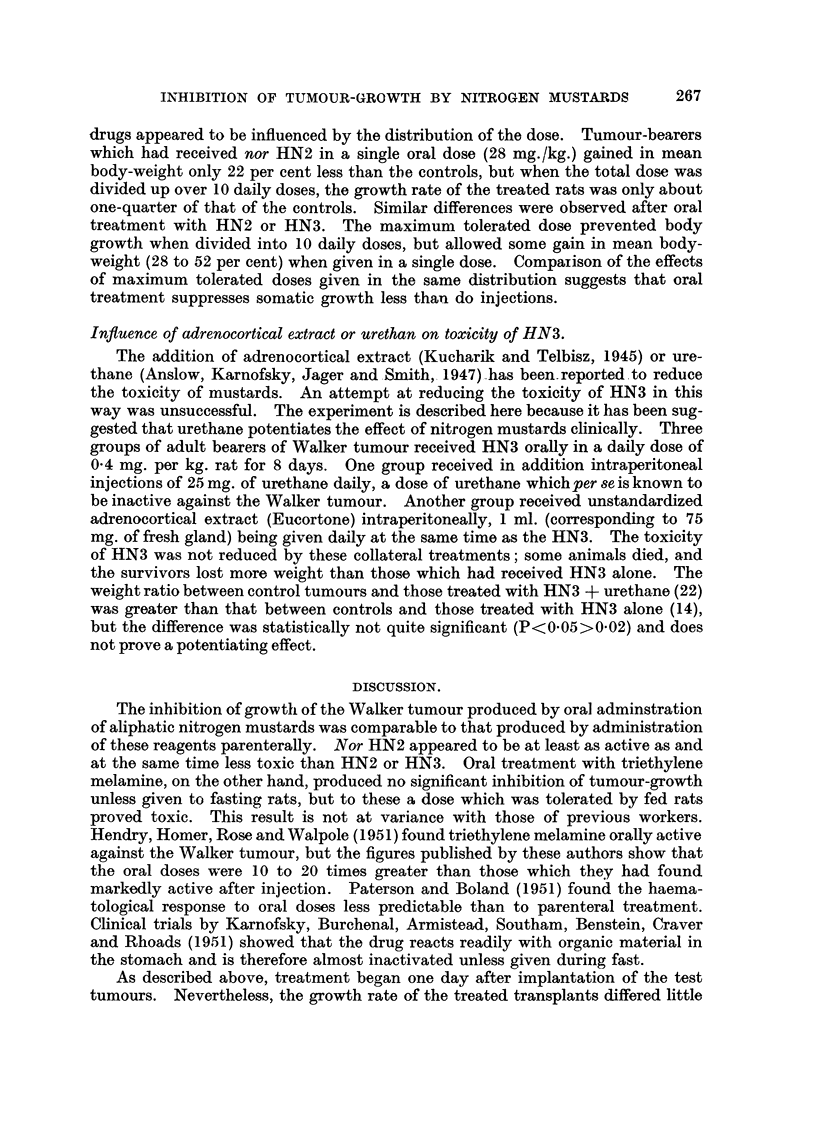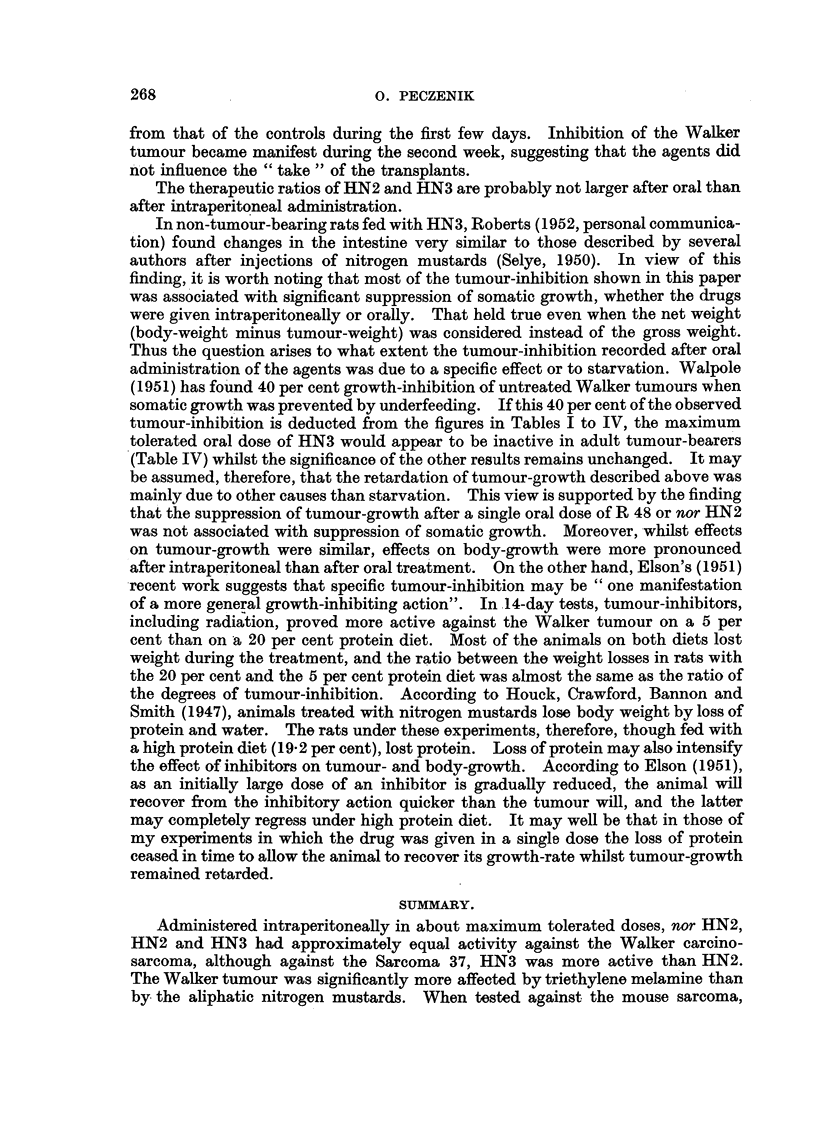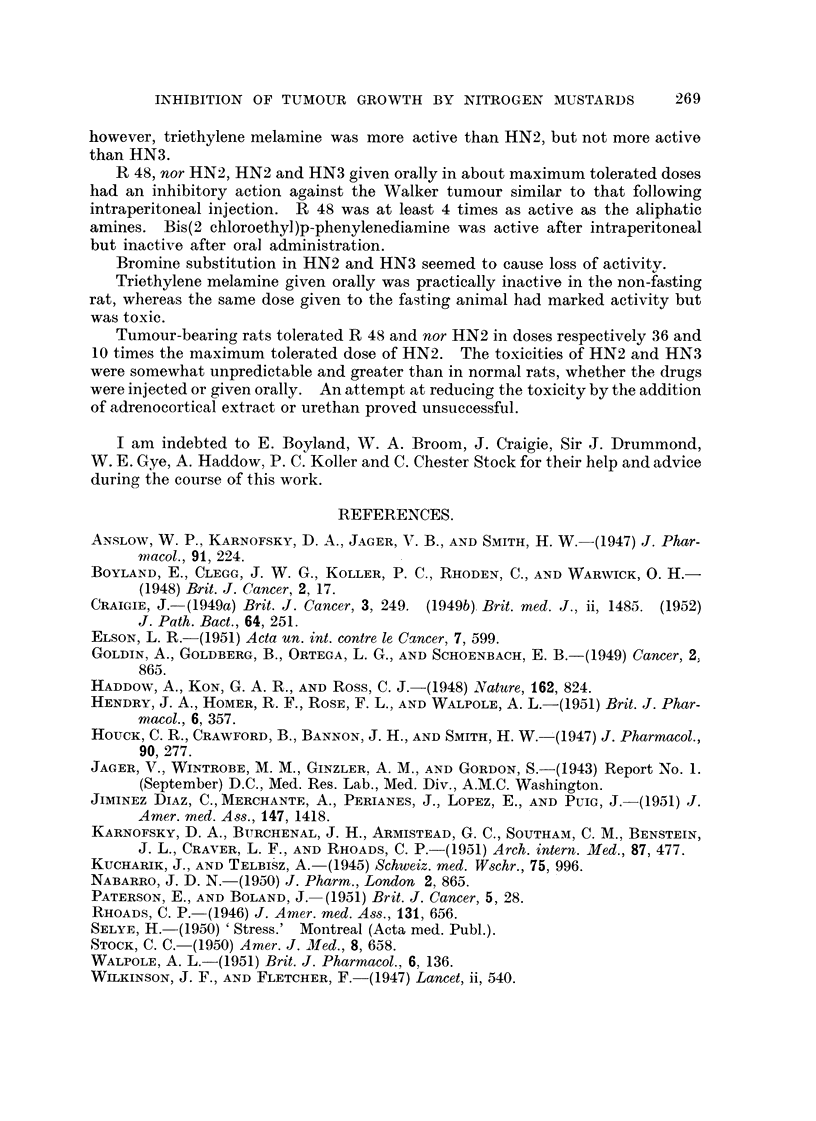# Inhibition of Tumour-growth After Oral and Parenteral Treatment with Nitrogen Mustards and Triethylene Melamine

**DOI:** 10.1038/bjc.1952.31

**Published:** 1952-09

**Authors:** O. Peczenik


### INHIBITION OF TUMOUR-GROWTH AFTER ORAL AND PARENTERAL TREATMENT WITH NITROGEN MUSTARDS AND TRIETHYLENE MELAMINE.

O. PECZENIK.

*From the Research Department (Pharmacology and Physiology Division), Boots Pure Drug Co. Ltd., Nottingham.*

Received for publication July 19, 1952.

INJECTIONS of aliphatic nitrogen mustards (Boyland, Clegg, Koller, Rhoden and Warwick, 1948 ; Stock, 1950) have proved of therapeutic value, but sometimes produce clinically undesirable side effects (Nabarro, 1950). The tris(2-chloroethyl)amine (HN3), for instance, seemed very promising (Wilkinson and Fletcher, 1947 ; Jiminez Diaz, Merchante, Perianes, Lopez and Puig, 1951), but its use has been discouraged by the great risk of thrombosis at the site of injection (Rhoads, 1946 ; Nabarro, 1950). Clinical reports on the oral activity of an aromatic nitrogen mustard, NN-di(2-chloroethyl)β naphthylamine, R 48 (Haddow, Kon and Ross, 1948) and of triethylene melamine, T.E.M. (Karnofsky, Burchenal, Armistead, Southam, Benstein, Craver and Rhoads, 1951), therefore, aroused much interest.

Human trials on the hazards of water contamination carried out by the American Army Medical Centre indicated that aliphatic nitrogen mustards might be absorbed after oral administration : HN3 in a total dose of 15 to 18 mg. per man caused transitory nausea, vomiting, anorexia, and the number of W.B.Cs. dropped to 2300 (Jager, Wintrobe, Ginzler and Gordon, 1943). It seemed of interest, therefore, to see if aliphatic nitrogen mustards have an oral activity against tumour-growth comparable to that of R 48 or T.E.M. Tests with these two compounds and with three aliphatic nitrogen mustards, bis(2-chloroethyl)amine (*nor* HN2), bis(2-chloroethyl)methylamine (HN2) and HN3, against transplanted tumour are described in this paper.

### TECHNIQUE.

*Test tumours.*—Subcutaneous transplants of Walker carcinosarcoma and of mouse Sarcoma 37 were used. The Walker tumours were passages of tumour cell suspension in dextrose, prepared by Craigie's mincer technique (Craigie, 1949) and preserved at—70°C. (Craigie, 1949, 1952) at the laboratories of the Imperial Cancer Research Fund. The Walker tumour was transplanted by trocar and Sarcoma 37 by Craigie's mincer technique.

*Test animals.*—Two age groups of female rats were used as hosts for the Walker tumour :

(*a*) A group of 52 $\pm$ 3·6 g. body weight as recommended by Gye (personal communication) ; (at this age the regular oestrus cycle has not yet developed and individual variations in the growth rate of the tumours are comparatively small). This group will be referred to as " young " rats.

(*b*) A group of 87 $\pm$ 5·6 g. body weight as recommended by Boyland (personal communication). For convenience these rats will be referred to as "adult" rats.

Sarcoma 37 was grown in male mice of 18 to 22 g. body weight.

The animals were fed on Thomson's diet (19·2 per cent protein), the rats receiving carrots in addition. Treatment began one day after implantation of the tumour and was continued on consecutive days, Sunday excepted. The mouse sarcoma was treated daily for 7 days. In the tests against the Walker tumour the total dose was given over 7, 8 or 10 days, and also as a single dose (Boyland, Cleg, Koller, Rhoden and Warwick, 1948; Haddow, Kon and Ross, 1948). Eleven to fifteen animals were used for test.

The water-soluble drugs were dissolved in distilled water immediately before administration and given orally or intraperitoneally. The daily dose was given in 0·4 ml. of distilled water per 100 g. rat. The oral dose was administered by stomach-tube, and immediately afterwards 0·8 ml. of distilled water was passed through the tube to ensure that the whole dose was washed into the stomach. R 48 was given in a single oral dose in arachis oil. Control animals received the solvent by stomach-tube or remained untreated. The Walker tumours and Sarcomata 37 were dissected 14 and 9 days respectively after transplantation, fixed in Bouin's fluid and 70 per cent alcohol, dried between filter-paper, and weighed. The difference in weight between control and treated tumours was analysed statistically. The ratio, mean weight of control tumour divided by mean weight of treated tumours, was used as the measure of the activity of the test compound. Duplicate experiments were carried out at different dates.

### RESULTS.

*Intraperitoneal treatment.*

Given in approximately the maximum tolerated doses, both HN2 and HN3 showed similar suppressive activity against the Walker tumour. The mouse sarcoma, however, appeared to be more responsive to HN3. The Walker tumour was significantly more affected by T.E.M. than by HN2 or HN3; the weight ratio between controls and T.E.M. treated tumours was about 14 times that between controls and tumours injected with the two mustards. When tested against the mouse sarcoma, however, T.E.M. was significantly more active than HN2, but not more active than HN3 (Table I). The maximum tolerated dose of *nor* HN2 was about 10 times that of HN2. When injected in these doses the two compounds had similar activity against the Walker tumour (Table II).

TABLE I.—*Comparative Tumour-inhibitory Activity of Intraperitoneal Treatment with Nitrogen Mustards and Triethylene Melamine.*

| Agent. | Daily dose (mg./kg.B.W.). | Total dose (mg./kg.B.W.). | Test tumour. | Effect. Ratio. | Effect. Signif. (P.). |
|---|---|---|---|---|---|
| HN2 hydrochloride | 0·12 | 1·20 | Walker | 7·2 | <0·01 |
| | 0·50 | 3·50 | Sarcoma 37 | 2·2 | 0·01 |
| | 0·50 | 3·50 | | 2·3 | <0·01 |
| HN3 hydrochloride | 0·10 | 1·00 | Walker | 7·8 | <0·01 |
| | 0·50 | 3·50 | Sarcoma 37 | 7·5 | <0·01 |
| Triethylene melamine | 0·16 | 1·60 | Walker | 104·0 | <0·01 |
| | 0·25 | 1·75 | Sarcoma 37 | 4·6 | 0·01 |

The monofunctional ethyleneimine in a total dose of 10 mg. per kg. was inactive. NN-bis(2 chloroethyl)p-phenylene diamine hydrochloride in a total dose of 4 mg. per kg. had significant activity (ratio 5·4 ; P<0·01).

*Oral treatment.*

(a) *Test animals fed* ad libitum.—The three aliphatic nitrogen mustards when administered orally also retarded tumour-growth. The maximum tolerated oral dose was about twice the maximum tolerated intraperitoneal dose. Using these doses, both oral and parenteral treatment produced similar effects against the Walker tumour (Table II). The oral activity seemed to be influenced by the distribution of the total dose ; the weight ratio between control and HN2-treated tumours became more significant when the total dose was divided over 7 instead of over 10 daily doses. Boyland, Cleg, Koller, Rhoden and Warwick (1948) found that the parenteral activity of nitrogen mustards increased when the total dose was given as a single injection. When given in a single oral dose the effect of HN3 was negligible, whereas the activity of HN2 and *nor* HN2 seemed to be increased to a certain extent. The oral activity of *nor* HN2 seemed at least equal to that of HN2, and more pronounced than that of HN3, but even the highest weight ratios between control-tumours and those treated with the aliphatic compounds were only about one-quarter of the ratio recorded after treatment with R 48 (Table II). The other aromatic nitrogen mustard tested, NN-bis(2 chloroethyl)p-phenylene diamine hydrochloride, when given as a single oral dose of 4 mg. per kg. was inactive. Larger doses were active but toxic.

T.E.M., though strikingly suppressive after intraperitoneal injection, retarded the growth of the Walker tumour to a negligible extent when given orally. When the maximum tolerated dose of 4 mg. per kg. rat was divided over 10 days, no activity was seen. When the same amount was given in a single dose, tumour-growth was slightly but probably significantly retarded.

TABLE II.—*Tumour inhibitory Activity of Nitrogen Mustards and Triethylene Melamine Administered to Young Rats* (52 ± 3·6 *g.*).

| Agent. | Route. | Daily dose (mg./kg.B.W.). | Total dose (mg./kg.B.W.). | Effect. | |
|---|---|---|---|---|---|
| | | | | Ratio. | Signif. (P.). |
| HN2 hydrochloride | Intrap. | 0·12 | 1·2 | 7·2 | <0·01 |
| | Oral | 0·28 | 2·8 | 5·4 | <0·02>0·01 |
| | ,, | 0·40 | 2·8 | 11·9 | <0·01 |
| | ,, | 2·80 | 2·8 | 7·6 | <0·01 |
| *nor* HN2 hydrochloride | Intrap. | 1·20 | 12·0 | 8·1 | <0·01 |
| | Oral | 2·80 | 28·0 | 10·2 | <0·01 |
| | ,, | 28·00 | 28·0 | 12·1 | <0·01 |
| HN3 hydrochloride | Intrap. | 0·10 | 1·0 | 7·8 | <0·01 |
| | Oral | 0·20 | 2·0 | 6·5 | <0·01 |
| | ,, | 0·20 | 2·0 | 6·6 | <0·01 |
| | ,, | 2·00 | 2·0 | 2·3 | 0·02 |
| | ,, | 2·00 | 2·0 | 2·4 | <0·02>0·01 |
| R 48 | ,, | 100·00 | 100·0 | 52·4 | <0·01 |
| Triethylene melamine | Intrap. | 0·16 | 1·6 | 104·0 | <0·01 |
| | Oral | 0·20 | 2·0 | 2·0 | 0·20 |
| | ,, | 0·40 | 4·0 | 1·4 | 0·20 |
| | ,, | 4·00 | 4·0 | 3·6 | <0·02>0·01 |

(b) *Fasting test animals.*—As shown in Table II, a single oral dose of T.E.M. or HN3 was almost inactive, but the same dose given to tumour-bearers which had been kept fasting for 22 or 32 hours before and 4 hours after treatment was significantly active. This dose, though tolerated by non-fasting rats, was toxic to the fasting animals (Table III).

TABLE III.—*Influence of Fasting on the Oral Activity of Single Doses of Triethylene Melamine and Nitrogen Mustard (Young Rats of* 50 ± 3·7 *g. Body-weight).*

| Agent. | Dose (mg./kg. B.W.). | Test animal. | No. of survivors. | Change in body-weight (%). | Effect. Ratio. | Effect. Signif. (P.). |
|---|---|---|---|---|---|---|
| Triethylene melamine | 4·0 | Fed | 12/12 | +52 | 3·6 | <0·02>0·01 |
| | 4·0 | Fasting | 8/12 | +40 | 27·0 | <0·01 |
| HN3 hydrochloride | 2·0 | Fed | 12/12 | +31 | 2·4 | <0·02>0·01 |
| | 2·0 | Fasting | 12/14 | +33 | 8·6 | <0·01 |

*Influence of the age of the test animals.*

All the tests described above were carried out on young rats. Some further tests were performed with the adult group to find whether the permeability of the gastro-intestinal mucosa decreases with maturation. Some of the results varied with the age-group. After oral treatment with HN3 the weight ratios between control and treated tumours were greater in the young rats. In adult rats 2 mg. per kg. retarded tumour-growth slightly but significantly; 2·8 and 3·2 mg. per kg. produced an effect similar to that produced in the young animals by 2 and 2·8 mg. per kg. respectively. It is doubtful whether these differences were due to a greater permeability of the gastro-intestinal mucosa in the young rats, for after oral treatment with HN2 the ratios were greater in the adult rats, but not in the young rats (Table IV). Moreover, a difference in response between the two age groups was also found after intraperitoneal treatment: T.E.M., injected in a total dose of 1·6 mg. per kg., suppressed tumour-growth even more strikingly in

TABLE IV.—*Tumour-inhibitory Activity of Nitrogen Mustards and Triethylene Melamine Administered to Young and Adult Rats.*

| Agent. | Route. | Daily dose (mg./kg. B.W.). | Total dose (mg./kg. B.W.). | Effect young rats. | | Effect adult rats. | |
|---|---|---|---|---|---|---|---|
| | | | | Ratio. | Signif. (P.). | Ratio. | Signif. (P.). |
| HN2 hydrochloride | Oral | 0·28 | 2·8 | 5·4 | <0·02>0·01 | 15·6 | <0·01 |
| | ,, | 0·40 | 2·8 | 12·1 | <0·01 | 25·3 | <0·01 |
| | ,, | 0·40 | 2·8 | | | 15·6 | <0·01 |
| HN3 hydrochloride | Intrap. | 0·10 | 1·0 | 7·8 | <0·01 | 1·7 | |
| | ,, | 0·10 | 1·0 | | | 8·6 | <0·01 |
| | Oral | 0·20 | 2·0 | 6·5 | <0·01 | 2·9 | <0·02>0·01 |
| | ,, | 0·20 | 2·0 | 6·6 | <0·01 | 3·5 | <0·01 |
| | ,, | 2·00 | 2·0 | 2·3 | 0·02 | 3·2 | <0·01 |
| | ,, | 2·00 | 2·0 | 2·4 | <0·02 | | |
| | ,, | 0·25 | 2·5 | | | 4·4 | <0·01 |
| | ,, | 0·40 | 2·8 | 13·9 | <0·01 | 7·2 | <0·01 |
| | ,, | 0·40 | 2·8 | 14·3 | <0·01 | | |
| | ,, | 0·40 | 3·2 | | | 14·0 | <0·01 |
| Triethylene melamine | Intrap. | 0·16 | 1·6 | 104·0 | <0·01 | 346·0 | <0·01 |
| | Oral | 0·40 | 4·0 | 1·4 | 0·2 | 1·6 | 0·1 |

the adult group ; the ratio was about 3 times that recorded in the young animals. Seven tests only were performed in duplicate, six of them against the Walker tumour. In the three tests with the young rats the duplicate results agreed, whilst in the three tests carried out with the adult rats the duplicates agreed in two but disagreed in the other (intraperitoneal treatment with HN3). In view of the smaller number of tests carried out in duplicate one cannot exclude the possibility that the difference in response between the two age-groups may represent only variations in susceptibility to the tumour-inhibitors between the individual batches of animals.

*Bromine substitutes.*

In the case of haloarylalkylamines with little or no tumour-inhibitory activity, it was found that the replacement of chlorine by the more reactive bromine produced active compounds (Haddow, Kon and Ross, 1948). Bromine substitution in HN2 and HN3, however, did not increase the activity against the Walker tumour ; in fact, given in the doses in which the chlorine compounds were active the two bromine compounds showed no inhibitory activity whatever.

*Toxicity of the nitrogen mustards and triethylene melamine in tumour-bearers.*

Given orally, R 48 was tolerated in a dose about 36 and 50 times the maximum tolerated doses of HN2 and HN3 respectively. The maximum tolerated oral dose of *nor* HN2 was about 10 times larger than that of HN2 and 14 times larger than that of HN3 (Table II). The therapeutic ratio of HN2 and HN3, whether the drugs were given intraperitoneally or orally, seemed as low as 2, or even lower. As estimated from the death-rate, HN2 and HN3 were more toxic to tumour-bearing than to normal rats. HN2, for example, in a daily intraperitoneal dose of 0·28 mg. per kg. was tolerated by normal rats for 18 days but proved toxic to tumour-bearerrs within 10 days. Oral treatment with HN3 in a daily dose of 0·28 mg. per kg. was tolerated by normal rats for 14 days, whilst 0·25 mg. per kg. was toxic to tumour-bearers after 10 days. Toxicity was somewhat unpredictable in tumour-bearers : in one test, for instance, injections of HN2 in daily doses of 0·27 mg. per kg. were tolerated, whilst in two others 0·2 mg. per kg. was toxic. HN3 given orally in a total dose of 3·2 mg. per kg. was tolerated by one batch of tumour-bearers, though individuals from other batches were killed by 2·5 mg. per kg.

Goldin, Goldberg, Ortega and Schoenbach (1949) demonstrated a similar variation in mice bearing Sarcoma 180 ; in four batches injected with HN2 (7·5 mg. per kg. mouse) mortality varied from zero to 25 per cent. The young rats were at least as resistant to toxicity as were the adults, and in some instances they seemed even more resistant. For instance, oral treatment with HN2 in 7 daily doses of 0·4 mg. per kg. was toxic to adult but tolerated by young tumour-bearers.

*Effect of nitrogen mustards and triethylene melamine on body-weight.*

The tumour-inhibitory effect of the agents investigated was frequently associated with inhibition of somatic growth. In some instances, more frequent in the adult than in the young rats, there was even loss of mean body-weight. Animals treated orally with R 48, T.E.M. or *nor* HN2 gained in body weight considerably more than those treated with HN2 or HN3. The stunting effect of the drugs appeared to be influenced by the distribution of the dose. Tumour-bearers which had received *nor* HN2 in a single oral dose (28 mg./kg.) gained in mean body-weight only 22 per cent less than the controls, but when the total dose was divided up over 10 daily doses, the growth rate of the treated rats was only about one-quarter of that of the controls. Similar differences were observed after oral treatment with HN2 or HN3. The maximum tolerated dose prevented body growth when divided into 10 daily doses, but allowed some gain in mean body-weight (28 to 52 per cent) when given in a single dose. Comparison of the effects of maximum tolerated doses given in the same distribution suggests that oral treatment suppresses somatic growth less than do injections.

*Influence of adrenocortical extract or urethan on toxicity of HN3.*

The addition of adrenocortical extract (Kucharik and Telbisz, 1945) or urethane (Anslow, Karnofsky, Jager and Smith, 1947) has been reported to reduce the toxicity of mustards. An attempt at reducing the toxicity of HN3 in this way was unsuccessful. The experiment is described here because it has been suggested that urethane potentiates the effect of nitrogen mustards clinically. Three groups of adult bearers of Walker tumour received HN3 orally in a daily dose of 0·4 mg. per kg. rat for 8 days. One group received in addition intraperitoneal injections of 25 mg. of urethane daily, a dose of urethane which *per se* is known to be inactive against the Walker tumour. Another group received unstandardized adrenocortical extract (Eucortone) intraperitoneally, 1 ml. (corresponding to 75 mg. of fresh gland) being given daily at the same time as the HN3. The toxicity of HN3 was not reduced by these collateral treatments; some animals died, and the survivors lost more weight than those which had received HN3 alone. The weight ratio between control tumours and those treated with HN3 + urethane (22) was greater than that between controls and those treated with HN3 alone (14), but the difference was statistically not quite significant ($P<0{\cdot}05>0{\cdot}02$) and does not prove a potentiating effect.

### DISCUSSION.

The inhibition of growth of the Walker tumour produced by oral adminstration of aliphatic nitrogen mustards was comparable to that produced by administration of these reagents parenterally. *Nor* HN2 appeared to be at least as active as and at the same time less toxic than HN2 or HN3. Oral treatment with triethylene melamine, on the other hand, produced no significant inhibition of tumour-growth unless given to fasting rats, but to these a dose which was tolerated by fed rats proved toxic. This result is not at variance with those of previous workers. Hendry, Homer, Rose and Walpole (1951) found triethylene melamine orally active against the Walker tumour, but the figures published by these authors show that the oral doses were 10 to 20 times greater than those which they had found markedly active after injection. Paterson and Boland (1951) found the haematological response to oral doses less predictable than to parenteral treatment. Clinical trials by Karnofsky, Burchenal, Armistead, Southam, Benstein, Craver and Rhoads (1951) showed that the drug reacts readily with organic material in the stomach and is therefore almost inactivated unless given during fast.

As described above, treatment began one day after implantation of the test tumours. Nevertheless, the growth rate of the treated transplants differed little from that of the controls during the first few days. Inhibition of the Walker tumour became manifest during the second week, suggesting that the agents did not influence the " take " of the transplants.

The therapeutic ratios of HN2 and HN3 are probably not larger after oral than after intraperitoneal administration.

In non-tumour-bearing rats fed with HN3, Roberts (1952, personal communication) found changes in the intestine very similar to those described by several authors after injections of nitrogen mustards (Selye, 1950). In view of this finding, it is worth noting that most of the tumour-inhibition shown in this paper was associated with significant suppression of somatic growth, whether the drugs were given intraperitoneally or orally. That held true even when the net weight (body-weight minus tumour-weight) was considered instead of the gross weight. Thus the question arises to what extent the tumour-inhibition recorded after oral administration of the agents was due to a specific effect or to starvation. Walpole (1951) has found 40 per cent growth-inhibition of untreated Walker tumours when somatic growth was prevented by underfeeding. If this 40 per cent of the observed tumour-inhibition is deducted from the figures in Tables I to IV, the maximum tolerated oral dose of HN3 would appear to be inactive in adult tumour-bearers (Table IV) whilst the significance of the other results remains unchanged. It may be assumed, therefore, that the retardation of tumour-growth described above was mainly due to other causes than starvation. This view is supported by the finding that the suppression of tumour-growth after a single oral dose of R 48 or *nor* HN2 was not associated with suppression of somatic growth. Moreover, whilst effects on tumour-growth were similar, effects on body-growth were more pronounced after intraperitoneal than after oral treatment. On the other hand, Elson's (1951) recent work suggests that specific tumour-inhibition may be " one manifestation of a more general growth-inhibiting action". In 14-day tests, tumour-inhibitors, including radiation, proved more active against the Walker tumour on a 5 per cent than on a 20 per cent protein diet. Most of the animals on both diets lost weight during the treatment, and the ratio between the weight losses in rats with the 20 per cent and the 5 per cent protein diet was almost the same as the ratio of the degrees of tumour-inhibition. According to Houck, Crawford, Bannon and Smith (1947), animals treated with nitrogen mustards lose body weight by loss of protein and water. The rats under these experiments, therefore, though fed with a high protein diet (19·2 per cent), lost protein. Loss of protein may also intensify the effect of inhibitors on tumour- and body-growth. According to Elson (1951), as an initially large dose of an inhibitor is gradually reduced, the animal will recover from the inhibitory action quicker than the tumour will, and the latter may completely regress under high protein diet. It may well be that in those of my experiments in which the drug was given in a single dose the loss of protein ceased in time to allow the animal to recover its growth-rate whilst tumour-growth remained retarded.

### SUMMARY.

Administered intraperitoneally in about maximum tolerated doses, *nor* HN2, HN2 and HN3 had approximately equal activity against the Walker carcinosarcoma, although against the Sarcoma 37, HN3 was more active than HN2. The Walker tumour was significantly more affected by triethylene melamine than by the aliphatic nitrogen mustards. When tested against the mouse sarcoma, however, triethylene melamine was more active than HN2, but not more active than HN3.

R 48, *nor* HN2, HN2 and HN3 given orally in about maximum tolerated doses had an inhibitory action against the Walker tumour similar to that following intraperitoneal injection. R 48 was at least 4 times as active as the aliphatic amines. Bis(2 chloroethyl)p-phenylenediamine was active after intraperitoneal but inactive after oral administration.

Bromine substitution in HN2 and HN3 seemed to cause loss of activity.

Triethylene melamine given orally was practically inactive in the non-fasting rat, whereas the same dose given to the fasting animal had marked activity but was toxic.

Tumour-bearing rats tolerated R 48 and *nor* HN2 in doses respectively 36 and 10 times the maximum tolerated dose of HN2. The toxicities of HN2 and HN3 were somewhat unpredictable and greater than in normal rats, whether the drugs were injected or given orally. An attempt at reducing the toxicity by the addition of adrenocortical extract or urethan proved unsuccessful.

I am indebted to E. Boyland, W. A. Broom, J. Craigie, Sir J. Drummond, W. E. Gye, A. Haddow, P. C. Koller and C. Chester Stock for their help and advice during the course of this work.

### REFERENCES.

Anslow, W. P., Karnofsky, D. A., Jager, V. B., and Smith, H. W.—(1947) *J. Pharmacol.*, **91**, 224.

Boyland, E., Clegg, J. W. G., Koller, P. C., Rhoden, C., and Warwick, O. H.—(1948) *Brit. J. Cancer*, **2**, 17.

Craigie, J.—(1949*a*) *Brit. J. Cancer*, **3**, 249. (1949*b*) *Brit. med. J.*, ii, 1485. (1952) *J. Path. Bact.*, **64**, 251.

Elson, L. R.—(1951) *Acta un. int. contre le Cancer*, **7**, 599.

Goldin, A., Goldberg, B., Ortega, L. G., and Schoenbach, E. B.—(1949) *Cancer*, **2**, 865.

Haddow, A., Kon, G. A. R., and Ross, C. J.—(1948) *Nature*, **162**, 824.

Hendry, J. A., Homer, R. F., Rose, F. L., and Walpole, A. L.—(1951) *Brit. J. Pharmacol.*, **6**, 357.

Houck, C. R., Crawford, B., Bannon, J. H., and Smith, H. W.—(1947) *J. Pharmacol.*, **90**, 277.

Jager, V., Wintrobe, M. M., Ginzler, A. M., and Gordon, S.—(1943) Report No. 1. (September) D.C., Med. Res. Lab., Med. Div., A.M.C. Washington.

Jiminez Diaz, C., Merchante, A., Perianes, J., Lopez, E., and Puig, J.—(1951) *J. Amer. med. Ass.*, **147**, 1418.

Karnofsky, D. A., Burchenal, J. H., Armistead, G. C., Southam, C. M., Benstein, J. L., Craver, L. F., and Rhoads, C. P.—(1951) *Arch. intern. Med.*, **87**, 477.

Kucharik, J., and Telbisz, A.—(1945) *Schweiz. med. Wschr.*, **75**, 996.

Nabarro, J. D. N.—(1950) *J. Pharm., London* **2**, 865.

Paterson, E., and Boland, J.—(1951) *Brit. J. Cancer*, **5**, 28.

Rhoads, C. P.—(1946) *J. Amer. med. Ass.*, **131**, 656.

Selye, H.—(1950) ' Stress.' Montreal (Acta med. Publ.).

Stock, C. C.—(1950) *Amer. J. Med.*, **8**, 658.

Walpole, A. L.—(1951) *Brit. J. Pharmacol.*, **6**, 136.

Wilkinson, J. F., and Fletcher, F.—(1947) *Lancet*, ii, 540.